# Impact of Origin and Biological Source on Chemical Composition, Anticholinesterase and Antioxidant Properties of Some St. John’s Wort Species (*Hypericum spp.*, Hypericaceae) from the Central Balkans

**DOI:** 10.3390/molecules181011733

**Published:** 2013-09-25

**Authors:** Biljana Božin, Nebojša Kladar, Nevena Grujić, Goran Anačkov, Isidora Samojlik, Neda Gavarić, Branislava Srđenović Čonić

**Affiliations:** 1 Department of Pharmacy, Faculty of Medicine, University of Novi Sad, Hajduk Veljkova 3, 21 000 Novi Sad, Serbia; E-Mails: nebojsa.kladar@gmail.com (N.K.); nevenagrujic@hotmail.com (N.Gr.); nedalakic@gmail.com (N.Ga.); srdjbr@yahoo.com (B.S.Č.); 2 Department of Biology and Ecology, Faculty of Sciences, University of Novi Sad, Trg D. Obradovića 2, 21 000 Novi Sad, Serbia; E-Mail: goran.anackov@dbe.uns.ac.rs; 3 Department of Pharmacology and Toxicology, Faculty of Medicine, University of Novi Sad, Hajduk Veljkova 3, 21 000 Novi Sad, Serbia; E-Mail: isidoras2011@gmail.com

**Keywords:** St. John’s wort, biological source, origin, composition, biological activities

## Abstract

The study shows the influence of the origin of plant material and biological source on the *in vitro* antioxidant (neutralization of DPPH and OH radical, nitric oxide, and inhibition of lipid peroxidation) and anticholinesterase activity of chemically characterized and quantified ethanol extracts of ten St. John’s wort samples. The investigated samples were: five *Hypericum perforatum* species representatives collected at different localities, one commercial sample of *Hyperici herba* purchased at a local market and four *Hypericum* species autochtonous to the Balkan Peninsula (*H. maculatum* subsp. *immaculatum*, *H. olympicum*, *H. richeri* subsp. *grisebachii* and *H. barbatum*). All the examined extracts exhibited notable antioxidant potential, but in most of the cases indigenous *Hypericum* species expressed stronger effects compared to the original source of the drug, *H. perforatum*. The changes in the content of phenolic compounds, especially flavonoids, hyperforin and hypericin, related to the source of the drug affected the investigated activities. Since all of the investigated species have shown prominent inhibition of acetylcholinesterase *in vitro* activity, they could be further investigated as potential substances in preventing of Alzheimer’s disease.

## 1. Introduction

St. John’s wort (*Hypericum perforatum* L., Hypericaceae) is a very popular and widely used traditional herbal remedy, accepted by conventional medicine, too. In different countries it is used mainly in the form of an oil macerate for the treatment of burns, bruises, eczema, dyspepsia and gastric ulcer, biliary disorders, inflammation of the bronchi and urogenital tract, common cold, migraine headaches and diabetes mellitus [1,2]. In the form of tea infusions or commercial herbal preparations (dry or liquid extracts, tinctures) it is used in the treatment of mild to moderate depressive episodes [3,4]. Besides its antidepressant activity, other pharmacological effects (antibacterial, antifungal and antiviral properties, relaxing smooth muscle contraction, inhibiting protein kinase C, potentiating wound healing, photodynamic effects) have also been experimentally confirmed [2,5].

In recent times, it has been established that depression can be present in patients suffering from Alzheimer’s disease (AD), since this condition is widely accompanied by cognitive abnormality and neurodegeneration [6,7]. One of the therapeutic opportunities to alleviate the symptoms present in AD is the inhibition of neuronal acetylcholinesterase activity [8]. Also, the antioxidant and anti-inflammatory activity of some preparations or isolated compounds is relevant for the treatment of AD, because the increased endogenous formation of free radicals can contribute to the formation of β-amyloid plaques and inflammation [9]. Several studies have showed notable anticholinesterase activity of *H. perforatum* [10,11,12] and some other *Hypericum* species extracts [13,14], but the activity have not been supported with the chemical composition or specific compounds identified in samples. However, one study [15] showed that hyperforin could prevent β-amyloid neurotoxicity and spatial memory impairments by disaggregation of β-amyloid deposits.

The crude drug (*Hyperici herba*) and commercial preparations have been well chemically investigated and the major constituents, responsible for pharmacological effects have been characterized [2,4]. Thus, for the registration of commercial phytopreparations, 0.1%–0.3% of naphtodianthrones (hypericin and its derivatives), up to 6% of phloroglucinol derivatives (expressed as a hyperforin) and 2%–4% of flavonoids (hyperoside) are required [4]. However, the quality of the drug, and consequently of phytopreparations, can vary due not only on the origin of the plant material, but also on environmental conditions and cultivar selection, as well as the subspecies collected [16,17,18,19]. Furthermore, the crude drug (*Hyperici herba*), which could be found on markets in many of the cities across the Balkans is not subjected to any quality control. Additional problems concerning the manipulation of original St. John’s wort (*H. perforatum*) crude drug or commercial pharmaceutical preparations by introducing indigenous/native *Hypericum* species which may not be morphologically similar or chemically equivalent. Although the presence of hyperforin and rutin is used to differentiate *H. perforatum* from other *Hypericum* species [2,20], recent investigations have showed that these two compounds are present, although in lower concentrations in several other representatives [10,21,22].

It is confirmed that compounds with phloroglucinol structure are mainly responsible for antimicrobial, antiviral and anticancer activity [23,24], but the controversy about the components responsible for the antidepressant effects is still topical. Early studies focused on the investigation of the MAO inhibitory effects of hypericin and other naphtodianthrones [25]. It has been conducted that the MAO inhibitory effect is connected with the presence of hyperforin [26]. Nowadays it has been indicated that an amentoflavone [27] or some other flavonoid derivatives [28] are the most likely to be responsible for the antidepressant activity of St. John’s wort.

Furthermore, many other species belonging to the genus *Hypericum* have long been known as traditional medicines, especially due to their strong antimicrobial and wound healing effects [29,30]. Some of these species, such as *H. venustum* [21], *H. undulatum* and *H. androsaemum* [10,31,32], and *H. rumeliacum* [30] have at least been partially chemically characterized. However, there is an increasing interest in the examination of different *Hypericum* species, especially related to the evaluation of antioxidant, antimicrobial, and recently found anticholinesterase activity [10,14,21,22,23,29,30,31,32,23,29]. Nevertheless, the studies of these species and their activity are often not supported with discovered quantification of the identified constituents [11,12,13,29]. Thus, investigation and quantification of the main bioactive compounds in extracts should be carried out, in order to correlate the plant constituency with potential activities.

In the present work have been evaluated the antioxidant effect, performed by different methods, *in vitro* inhibition of acethylcholinesterase activity and correlated with the chemical constituency of ethanol extracts of ten St. John’s wort samples (five *H. perforatum* subsp. *perforatum* collected from different localities, one trade sample of *Hyperici herba* purchased at a local market and four *Hypericum* species autochthonous to the Balkan peninsula—*H. maculatum* subsp. *immaculatum*, *H. olympicum*, *H. richeri* subsp. *grisebachii* and *H. barbatum*). Results represent the first report of the influence of autochthonous Balkan species on acetylcholinesterase activity and a comprehensive review on the influence of the origin on the quality of the extracts and bioactivity of common St. John’s wort (*Hypericum perforatum*).

## 2. Results and Discussion

### 2.1. Chemical Characterization of Examined Hypericum Extracts

Most antioxidant and some other biological/pharmacological activities from different plant sources are derived from phenolic-type compounds [33,34]. These effects do not always correlate with the presence of specific phenolics in large quantities, especially when a subclass of flavonoids and/or naphtodianthrones is in qualition. Therefore, quantification of compounds possibly useful for taxonomic determination, and designation of the biological origin of the drug are both necessary and need to be examined together. Regarding the above mentioned, the investigated extracts have been analyzed for the yield of dry extract (de), the total phenolic and flavonoid content, as well as for the quantity of the selected compounds considered to be responsible for most of St. John’s wort pharmacological activities [2].

The amount of total phenolics has varied widely in the examined extracts and ranged from 13.66 (*H. richeri*) to 30.36 mg GAE/g de (*H. barbatum*) (Table 1). Furthermore, the results obtained from the evaluation of the total flavonoid content have also pointed out great variations (1.33 for *H. perforatum* sample 4—6.74 mg QE/g de in *H. olympicum*), especially regarding the plant source. Notable differences between the content of total phenolics and flavonoids have been recorded and followed by differences in the yield of dry extract in the collected plant material (14.59%–52.63%) (Table 1). The obtained results for the yield of dry extract are mainly in correlation with the earlier published data on *Hypericum perforatum* [10], but also on some other investigated species [29]. However, in the most of the publications cited the completed taxonomical indentification of the examined *Hypericum* species is not provided [10,11,29,35,36], so the detail comparision of the obtained results was not possible to perform.

**Table 1:** Content of dry extract (g/100g of crude drug), total phenolics (mg GAE/g de) and total flavonoids (mg QE/g de) in the extracts of investigated species of *Hypericum*.

Source	Dry extract yeald (%)	Total phenolics (mg GAE/g de)	Total flavonoids (mg QE/g de)
1 (*H. perforatum*)	16.91 ± 0.09	15.49 ± 0.25	1.90 ± 0.03
2 (*H. perforatum*)	22.69 ± 0.32	14.65 ± 0.54	1.54 ± 0.05
3 (*H. perforatum*)	25.96 ± 0.51	16.72 ± 0.70	2.48 ± 0.06
4 (*H. perforatum*)	14.59 ± 0.27	15.33 ± 0.48	1.33 ± 0.04
5 (*Hyperici herba*)	21.04 ± 0.40	19.41 ± 0.32	2.85 ± 0.02
6 (*H. perforatum*)	19.29 ± 0.60	14.35 ± 0.54	2.23 ± 0.05
7 (*H. maculatum*)	16.79 ± 0.13	15.41 ± 0.19	5.51 ± 0.11
8 (*H. richeri*)	21.83 ± 0.32	13.66 ± 0.80	2.77 ± 0.04
9 (*H. olympicum*)	21.96 ± 0.47	16.16 ± 0.25	6.74 ± 0.08
10 (*H. barbatum*)	52.63 ± 0.12	30.36 ± 0.87	5.68 ± 0.07

Determination of the yield of dry extract, the total phenolic and flavonoid content in the extracts obtained from *H. perforatum* collected at different localities, and one trade sample (*Hyperici herba*) has also involved great variations. Thus, the content of dried extract in the plant material has varied from 14.59% to 25.96%, total phenolics have been in the range from 14.35 to 19.41 mg of GAE/g de, and the content of total flavonoids have ranged from 1.33 to 2.85 mg of QE/g de (Table 1). These divergences in the obtained results could be at least partially explained with the content of specific subclasses of phenolics such as phenolic acids, flavonoids, phloroglucinol compounds, naphtodianthrones and tannins in the examined St. John’s wort extracts [15,21,35,36]. However, the highest content of total phenolics and flavonoid compounds in the sample purchased at the local market (*Hyperici herba*) could point to the mixture of at least two species, possibly of *H. perforatum* and of some similar *Hypericum* species that flourish at the named locality at the same time.

**Figure 1:**
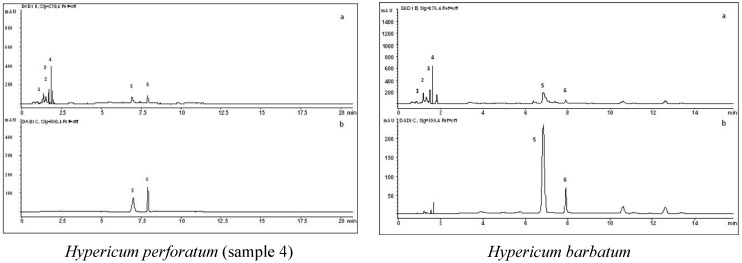
Sample chromatograms for *Hypericum perforatum* (sample 4) and *Hypericum barbatum*, with detection at 270 (**a**) and 590 nm (**b**) (1-chlorogenic acid, 2-caffeic acid, 3-rutin, 4-quercitrin, 5-hyperforin, 6-hypericin).

The specific compounds, mostly assumed to be responsible for the wide range of pharmacological activities, have been identified and quantified and the results are presented in the Table 2. The obtained HPLC chromatogram profiles of the investigated extracts (Figure 1) have been similar to those previously reported for ethanol extracts [16,21,35,36] of *H. perforatum* and other investigated *Hypericum* species. However, some differences related to the used solvent (water, methanol, supercritical carbon dioxide) were previously found [10,16,21].

**Table 2:** Phenolic composition of investigated *Hypericum* species extracts (HPLC analysis).

	Percentage (%) of identified compounds (mg/100mg de)					
Source	Phenolic acids		Flavonoids		Phloroglucinols	Naphtodianthrones
	Chlorogenic	Caffeic	Rutin	Quercitrin	Hyperforin	Hypericin
1 (*H. perforatum*)	0.38 ± 0.02	0.06 ± 0.005	0.33 ± 0.05	0.09 ± 0.004	0.78 ± 0.11	0.72 ± 0.09
2 (*H. perforatum*)	0.37 ± 0.04	0.07 ± 0.002	0.34 ± 0.02	0.11 ± 0.009	0.76 ± 0.07	0.56 ± 0.07
3 (*H. perforatum*)	0.44 ± 0.01	0.07 ± 0.001	0.36 ± 0.04	0.12 ± 0.008	1.71 ± 0.09	0.98 ± 0.11
4 (*H. perforatum*)	0.65 ± 0.03	n.d.	0.66 ± 0.07	0.19 ± 0.006	1.41 ± 0.12	1.11 ± 0.13
5 (*Hyperici herba*)	0.49 ± 0.05	0.08 ± 0.003	0.39 ± 0.03	0.15 ± 0.005	0.66 ± 0.08	0.59 ± 0.08
6 (*H. perforatum*)	0.38 ± 0.02	0.06 ± 0.002	0.34 ± 0.05	0.09 ± 0.003	0.91 ± 0.07	0.67 ± 0.05
7 (*H. maculatum*)	1.00 ± 0.04	0.17 ± 0.006	0.90 ± 0.09	0.25 ± 0.011	n.d.	n.d.
8 (*H. richeri*)	1.30 ± 0.06	0.07 ± 0.003	0.34 ± 0.02	0.09 ± 0.004	0.71 ± 0.09	n.d.
9 (*H. olympicum*)	0.38 ± 0.01	0.06 ± 0.001	0.32 ± 0.04	0.10 ± 0.002	1.82 ± 0.11	0.41 0.09
10 (*H. barbatum*)	0.49 ± 0.05	0.10 ± 0.004	0.39 ± 0.06	0.23 ± 0.009	2.97 ± 0.15	0.91 ± 0.11
Retention Times	1.39	1.55	1.67	1.80	6.83	7.91
Trendline equation	y = 6595.3x + 56.447	y = 11261x − 50.639	y= 453.2x − 105.2	y = 18924x − 55.18	y = 5473.7x − 14.86	y = 5572x − 3.0979
R-Squared value	R^2^ = 0.9985	R^2^ = 0.9975	R^2^ = 0.9974	R^2^ = 0.9991	R^2^ = 0.9989	R^2^ = 0.9973

n.d.—not detectable.

Among the identified compounds, the grouping of subclasses has been observed, with phenolic acids (chlorogenic and caffeic) and flavonoids (rutin and quercitrin) eluting between 0 and 2 min. The second group consists of phloroglucinols and naphtodiantrhrones (peaks appearing between 6.5 and 8 min). In the examined extracts notable variability of quantified compounds has been observed, both between the investigated *Hypericum* species and among the collected St. John’s wort samples. Especially important is the effect of variation of the pharmacologically most important compounds—hypericin (from not detectable in *H. maculatum* and *H. richeri* to 1.11% in *H. perforatum* collected on Cer Mt.) and hyperforin (from not detectable in *H. maculatum* to 2.97% in *H. barbatum*). Similar results have also been recorded also for other quantified compounds. The obtained results are in accordance with the earlier published data for the presence or the content of listed compounds [35,36].

In order to determine the differences between investigated extracts, Principle Compound Analysis (PCA) has been used. This analysis allows the overwiev of the complete variability of the examined extracts, and the number of original variables narrows to a smaller number (eigenvalues).

**Figure 2:**
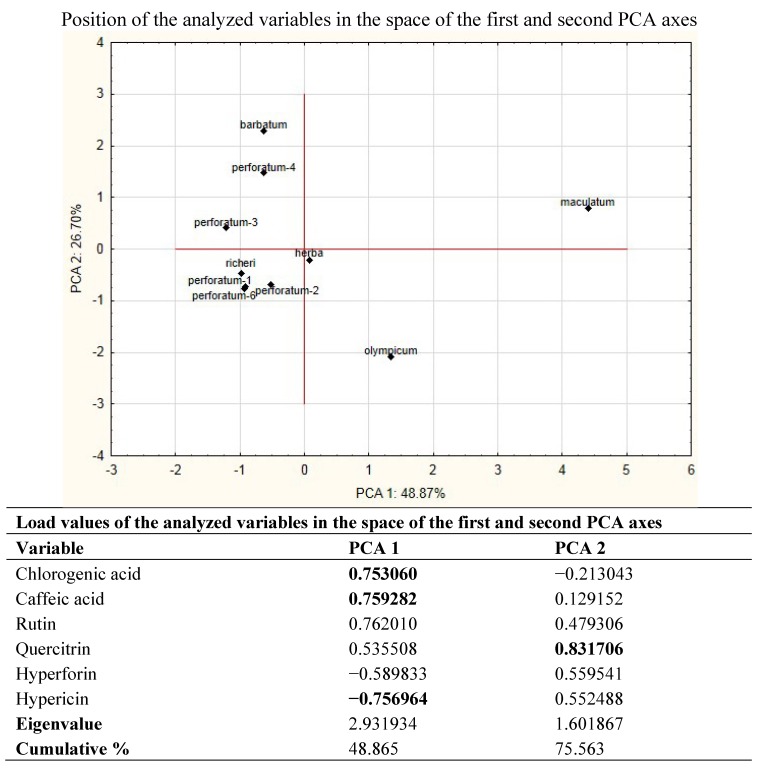
PCA based on compounds of investigated *Hypericum* species detected by HPLC analysis.

The analysis of the identified components using PCA pointed on six major vectors, of which the first two describe more than 75% of the total variability. Factors that contribute most to the separation of the extracts are the presence or amount of chlorogenic acid, hypericin and hyperforin (Figure 2). Drastic separation of *H. maculatum* subsp. *immaculatum* from all other investigated *Hypericum* representatives is primarily a consequence of the absence of hypericin and hyperforin, but also the result of highest content of chlorogenic and caffeic acid and rutin in this extract. In addition, the position of *H. olympicum* in the positive zone of first axis is the result of specific relationships in the values of all analyzed components and the presence of significant amounts of hyperforin.

In the space defined by the second axis, the main factor of the discrimination is the concentration of quercitrin, which is by the using of PCA proved to be unstable marker for distinguishing of individual *Hypericum* species, and its content in the plant is probably ecologically defined (Table 2). The central position of *Hyperici herba* (Figure 2) confirms the previous assumption that it is a mixture of at least two species, especially those with a significant amount of chlorogenic acid and rutin (e.g., *H. maculatum*), but also those with high content of hypericin and hyperforin, which are the caracteristic compounds of *H. perforatum*.

### 2.2. Antioxidant Activity

Antioxidant potential of different plant extracts and pure compounds has recently become the most assayed activity. Studies have showed that plants possess a wide range of compounds which exhibit significant potential for neutralizing free radicals and inhibiting the process of lipid peroxidation. This activity can be measured using numerous *in vitro* assays. However, due to their complex composition, a single method [10,29] is not recommended for the evaluation of antioxidant properties of different plant products [33,35,36]. Especially the DPPH assay is not a convenient single method for determination of an activity, because it is a synthetic free radical and does not exist in Nature. Furthermore, this system is best suitable for extracts prepared in organic solvents. Also, water extracts normally produce bubbles in the system and spectrophotometric readings are not entirely valid. On contrary, extracts which are not active using DPPH, may be more active using other systems.

Nevertheless, it is one of the most commonly used substrates for fast *in vitro* evaluation of the antioxidant activity due to its stability in the radical form and simplicity of the assay. Therefore, the antioxidant effects of plant products must be evaluated by combining at least two or more different *in vitro* assays to get relevant data. Thus, antioxidant properties of the examined *Hypericum* extracts have been evaluated, both for its free radical scavenging capacity (RSC) on a series of free radicals and for its ability to inhibit lipid peroxidation.

For the preliminary screening of the antioxidant capacity all the examined extracts were subjected to the DPPH assay. This method evaluates the ability of the investigated samples (either extracts or isolated substances) to act as donors of hydrogen atoms or electrons in transformation of DPPH radical into its reduced form, DPPH-H.

All the assessed extracts were able to reduce the stable, purple coloured radical DPPH into the yellow-coloured DPPH-H in dose dependent manner, reaching 50% of reduction. The IC_50_ values for *H. perforatum* and *Hyperici herba* (Table 3) ranged from 1.36 to 5.68 μg/mL and they are in accordance with the other published data [10,35]. Similar results have been obtained for the other examined *Hypericum* species (Table 4). However, these results are not so comparable with the other published results, because different units have been used for data expression [23,36].

Among all the investigated samples, the most powerful neutralization of DPPH radical was exhibited by the extract obtained from the material purchased at the local market (*Hyperici herba*) (Table 3). This result is in direct correlation with the content of total phenolics and flavonoids, which were notably higher in comparison with the extracts obtained from *H. perforatum* (Table 1). Comparison of the IC_50_ value (1.36 μg/mL) of the extract from the purchased herbal material with those obtained in all the other assayed extracts, together with determination of total phenolics and flavonoids, can additionaly point to the collectors confusion of *H. perforatum* with other indigenous *Hypericum* species or to mixture of at least two species.

**Table 3:** Antioxidant potential of examined *H. perforatum* and *Hyperici herba* extracts.

	Source					
	1 (*H. perforatum*)	2 (*H. perforatum*)	3 (*H. perforatum*)	4 (*H. perforatum*)	5 (*Hyperici herba*)	6 (*H. perforatum*)
	Free radical scavenging capacity (IC_50_ values presented in μg/mL)					
DPPH (IC_50_ values)	3.48 ± 0.08	3.82 ± 0.11	3.61 ± 0.07	4.26 ± 0.12	1.36 ± 0.05	5.68 ± 0.14
Trendline equation	y = 13.87x + 1.656	y = 12.98x + 0.402	y = 12.34x + 5.429	y = 11.50x + 1.032	y = 22.27ln(x) + 43.19	y = 7.948x + 4.820
R-Squared value	R^2^ = 0.993	R^2^ = 0.995	R^2^ = 0.990	R^2^ = 0.988	R^2^ = 0.989	R^2^ = 0.983
OH (IC_50_ values)	43.07 ± 0.16	59.40 ± 0.09	66.95 ± 0.13	>70.00	54.57 ± 0.10	53.04 ± 0.08
Trendline equation	y = 14.032ln(x) − 2.8004	y = 12.561ln(x) − 1.3034	y = 11.657ln(x) + 0.9948	y = 13.879ln(x) − 10.677	y = 0.4812x + 23.742	y=10.256ln(x) + 9.272
Squared value (R^2^)	R^2^ = 0.9968	R^2^ = 0.9244	R^2^ = 0.9859	R^2^ = 0.9938	R^2^ = 0.9696	R^2^ = 0.9616
NO (IC_50_ values)	92.82 ± 0.21	127.03 ± 0.19	143.31 ± 0.25	>170.00	112.32 ± 0.16	96.76 ± 0.17
Trendline equation	y=16.486ln(x) − 24.693	y=23.363ln(x) − 63.18	y=22.607ln(x) − 62.244	y=20.475ln(x) − 57.144	y= 25.057ln(x) − 68.302	y=26.585ln(x) − 71.552
Squared value (R^2^)	R^2^ = 0.9905	R^2^ = 0.9698	R^2^ = 0.9545	R^2^ = 0.9601	R^2^ = 0.9519	R^2^ = 0.9458
	Inhibition of LP (IC_50_ values presented in μg/mL)					
LP(IC_50_ values)	29.48 ± 0.07	74.06 ± 0.22	14.81 ± 0.12	36.77 ± 0.17	50.84 ± 0.11	45.64 ± 0.19
Trendline equation	y = 19.50ln(x) − 15.99	y= 23.80ln(x) − 52.48	y =23.07ln(x) − 12.18	y = 19.13ln(x) − 18.97	y = 13.42ln(x) − 2.726	y = 1.051x + 1.991
Squared value (R^2^)	R^2^ = 0.955	R^2^ = 0.977	R^2^ = 0.995	R^2^ = 0.939	R^2^ = 0.973	R^2^ = 0.990

**Table 4:** Antioxidant potential of *H. maculatum*, *H. richeri*, H. *olympicum* and *H. barbatum* extracts.

	**Source**			
	7 (*H. maculatum*)	8 (*H. richeri*)	9 (*H. olympicum*)	10 (*H. barbatum*)
	**Free radical scavenging capacity (IC_50_ values presented in μg/mL)**			
**DPPH (IC_50_ values)**	**4.10 ± 0.05**	**5.01 ± 0.07**	**3.80 ± 0.04**	**2.91 ± 0.09**
**Trendline equation**	y = 10.59x + 6.542	y = 10.39x − 2.037	y = 11.74x + 5.341	y = 17.34x − 0.519
**R-Squared value**	R^2^ = 0.982	R^2^ = 0.977	R^2^ = 0.994	R^2^ = 0.993
**OH (IC_50_ values)**	**70.09 ± 0.21**	**42.90 ± 0.15**	**59.74 ± 0.18**	**62.35 ± 0.25**
**Trendline equation**	y = 11.881ln(x) − 0.4923	y = 14.056ln(x) − 2.8353	y = 12.77ln(x) − 2.2295	y = 11.616ln(x) + 1.9937
**Squared value (R^2^)**	R^2^ = 0.9642	R^2^ = 0.961	R^2^ = 0.9501	R^2^ = 0.9871
**NO (IC_50_ values)**	**112.23 ± 0.24**	**155.73 ± 0.29**	**201.09 ± 0.22**	**79.62 ± 0.19**
**Trendline equation**	y = 25.647ln(x) − 71.067	y = 23.101ln(x) − 66.617	y = 0.2719x − 4.6752	y = 16.855ln(x) − 23.779
**Squared value (R^2^)**	R^2^ = 0.951	R^2^ = 0.9508	R^2^ = 0.9592	R^2^ = 0.9595
	**Inhibition of LP (IC_50_ values presented in μg/mL)**			
**LP(IC_50_ values)**	**29.90 ± 0.13**	**31.81 ± 0.09**	**39.46 ± 0.11**	**14.57 ± 0.07**
**Trendline equation**	y = 29.84ln(x) − 51.40	y = 26.24ln(x) − 40.80	y = 19.66ln(x) − 22.27	y = 19.04ln(x) − 1.023
**Squared value (R^2^)**	R^2^ = 0.995	R^2^ = 0.977	R^2^ = 0.977	R^2^ = 0.969

In living organisms hydroxyl radical can damage almost all types of macromolecules, carbohydrates, lipids, nucleic and amino acids, and lead to development of many diseases caused by oxidative stress. Unlike superoxide anion radical, which can be detoxified from the organism by superoxide dismutase, the OH radical cannot be eliminated by any enzymatic reaction, but only by action of some non-enzymatic endogenous or dietary antioxidants [37]. The ability of the examined *Hypericum* extracts to scavenge hydroxyl radical is shown in Table 3 and Table 4. Similarly to the results obtained in the DPPH-assay, all the examined extracts have exhibited ability to scavenge the OH radical in dose dependent manner. Values that represent the 50% of neutralization are in the range from 42.90 (*H. richeri* subsp. *grisebachii*) (Table 4) to > 70.00 μg/mL (*H. perforatum* collected on the mountain of Cer) (Table 3). However, the results cannot be compared with some others, because there is no available information on this activity for different *Hypericum* species.

In vertebrates, nitric oxide (NO) is an important signalling molecule involved in many physiological processes, playing a role in a wide range of biological responses and pathways. On the other hand, it can lead to development of some pathological processes. The toxicity of NO is attributed to its ability to bind to proteins that contain heme, iron or copper, which results in protein disruption. In reaction with a protein, NO can be either oxidized (lose electrons) or reduced (gain electrons). This ability is further responsible for the high reactivity of free radicals [38]. The results which indicate the examined extracts potential to reduce the nitric oxide formation are presented in Table 3 and Table 4. All the assayed extracts have exhibited ability to scavenge NO radical spontaneously released from sodium nitroprusside, but the results have varied from 79.62 (*H. barbatum*) to 201.09 μg/mL (*H. olympicum*) (Table 4). Similarly to the previous tests, significant differences in the results between the *Hypericum perforatum* extracts (Table 4) have been obtained and they are comparable with the other published data [35,36]. It can be seen from the data presented in Table 3 and Table 4 that a great variability in NO scavenging activity relates to the origin of the plant material (Table 5). For the other investigated species there are still no data sources available.

The protective role of the examined *Hypericum* extracts on the process of lipid peroxidation has been evaluated by the TBA-assay using Fenton reaction (Fe^2+^/H_2_O_2_) as an induction system. The inhibition of LP was determined by measuring the formation of MDA, using commercial corn oil as an oxidable substrate. In Table 3 and Table 4 is presented the ability of the examined *H. perforatum* and *Hyperici herba* extracts to inhibit the LP. All the extracts have exhibited notable inhibitory potential, with IC_50_ values ranging from 14.81 to 74.06 μg/mL. For *H. perforatum* the obtained results (Table 3) are similar to those published by Silva *et al.* [36]. However, in most of the cases the 50% of LP inhibition is notably lower than those values published for inhibition of LP in various fractions of *H. perforatum* extract [35]. The other examined *Hypericum* extracts (*H. maculatum* subsp. *immaculatum*, *H. olympicum*, *H. richeri* subsp. *grisebachii* and *H. barbatum*) have exhibited different antioxidant activities (IC_50_ values ranging from 14.57 to 39.46 μg/mL) (Table 4). Furthermore, the extract obtained from *H. barbatum* has expressed the strongest protective effect on the process of lipid peroxidation. Generally, all the four examined indigenous species have exhibited notably higher antioxidant capacity than those obtained for most of the investigated extracts of *H. perforatum*. Similarly to the OH radical, these results cannot be compared with some others, because there is no available information on this activity for different *Hypericum* species.

### 2.3. Inhibition of Acetylcholinesterase Activity

**Figure 3:**
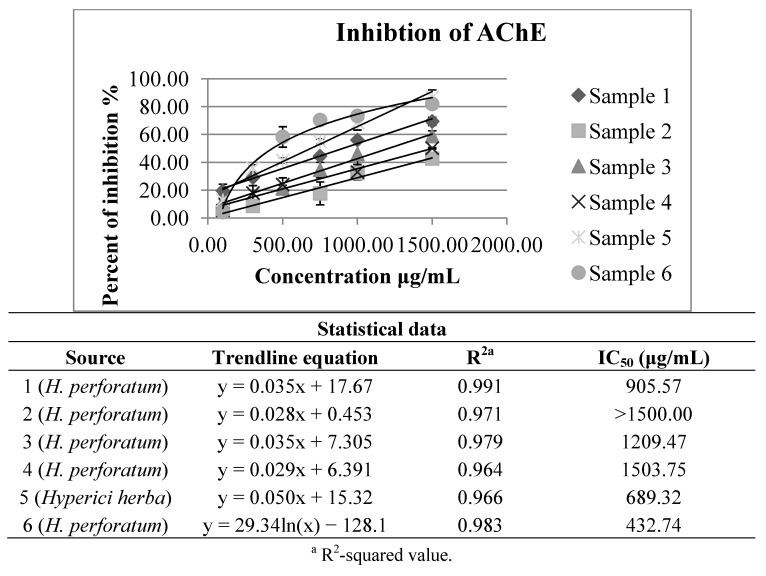
Inhibition of acetylcholinesterase by investigated *H. perforatum* extracts and by that obtained from *Hyperici herba*.

The results regarding anticholinesterase activity of the five *Hypericum perforatum* extracts and one obtained from the material purchased at a local market are shown in Figure 3. All of them have exhibited notable inhibition of acetylcholinesterase activity, with IC_50_ values between 432.74 and over 1500.00 μg of dry extract per mL. These results are in agreement with the previously published data [10,11].

**Figure 4:**
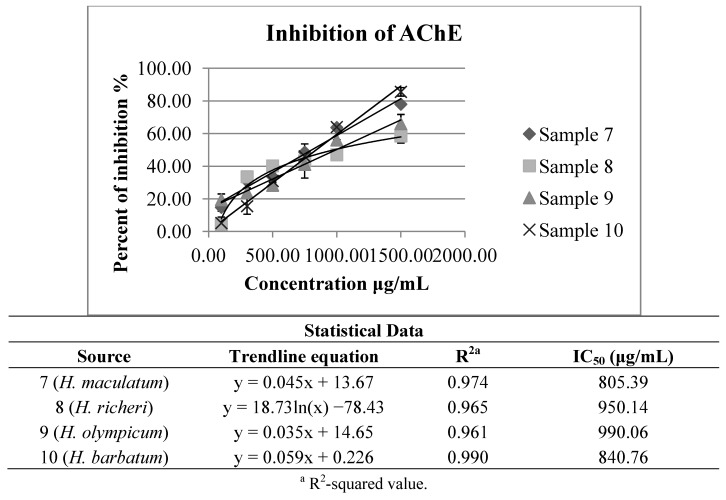
Inhibition of acetylcholinesterase by investigated *H. maculatum* subsp. *immaculatum*, *H. olympicum*, *H. richeri* subsp. *grisebachii* and *H. barbatum* extracts.

The IC_50_ values of anticholinesterase activity obtained for the other investigated *Hypericum* species (*H. maculatum* subsp. *immaculatum*, *H. olympicum*, *H. richeri* subsp. *grisebachii* and *H. barbatum*) (Figure 4) have been around the mean values of the results obtained for *H. perforatum* extracts (Figure 3). Although in most the cases notably stronger effects on the inhibition of acetylcholinesterase have been exhibited, for these autochthonous species of *Hypericum* there are still no available data on the studied activity, either.

## 3. Experimental

### 3.1. Plant Material

The complete list of plant material, with taxonomical information, voucher numbers, locality and collection period is shown in Table 5. All the voucher specimens are deposited in the BUNS Herbarium (Herbarium of the Department of Biology and Ecology, Faculty of Natural Sciences and Mathematics, University of Novi Sad). Also, the taxonomic placements are given within the sections on the genus *Hypericum* [39,40,41]. All the specimens and the plant material prepared for the examinations were collected at the full blossom stage.

### 3.2. Chemicals

The following HPLC grade compounds were used as standards for the analysis by HPLC-DAD: quercetin, caffeic acid, chlorogenic acid, quercitrin, rutin, hyperforin and hypericin (Extrasynthese, Genay Cedex, France). Folin-Ciocalteu (FC) reagent and HPLC grade methanol (MeOH) were purchased from Merck (Darmstadt, Germany). Analytical grade ethanol (EtOH) was obtained from Zorka pharma (Šabac, Serbia).Gallic acid, 2-thiobarbituric acid (TBA) and 2-deoxyribose were from Sigma Aldrich (St. Louis, MO, USA). Sodium bicarbonate, ferium sulphate and MeOH (analytical grade) were from POCH (Gliwice, Poland). Tween 80, 5,5'-dithiobis-(2-nitrobenzoic acid) – DTNB and acetonitrile (HPLC grade) were from J.T. Baker (Arlington Heights, IL, USA). Hydrogen peroxide, amonium acetate and formic acid were purchased from Lach-ner (Brno, Czech Republic). Acetylthiocholine iodide, N-(1-naphthyl) ethylenediamine dihydrochloride (NEDA), salicylamide (SA) and 2,2-diphenyl-1-pycrylhydrazil (DPPH) were from Alfa Aesar (Karlsruhe, Germany). Sodium nitropruside (SNP) was from Centrohem (Stara Pazova, Serbia). Acetylcholinesterase solution was from Roche (Basel, Switzerland). Corn oil was from Uvita (Debeljača, Serbia).

**Table 5:** Data relevant to examined *Hypericum* species collected during July 2012.

Sample No.	Section	Plant species	Locality	Voucher number (BUNS)
1	*Hypericum*	*H. perforatum* L. subsp. *perforatum*	Nidže, Mt., Skočivir-Mala reka F.Y.R. Macedonia	2-1746
2		*H. perforatum* L. subsp.	Mariovo; Moravik	2-1745
		*perforatum*	F.Y.R. Macedonia	
3		*H. perforatum* L. subsp.	Bistra Mt., Medenica;	2-1744
		*perforatum*	F.Y.R. Macedonia	
4		*H. perforatum* L. subsp.	Cer Mt.	2-1743
		*perforatum*	NorthWest Serbia	
5		*Hyperici herba*, trade sample	Stara planina Mt. East Serbia	2-1742
6		*H. perforatum* L. subsp.	Padej, Vojvodina	2-1741
		*perforatum*	North Serbia	
7		*H. maculatum* Crantz subsp. *immaculatum* (Murb.) A. Fröhlich	Korab Mt., Kobilino pole, F.Y.R. Macedonia	2-1739
8	*Olympia* (Spach) Nyman	*H. olympicum* L.	Mariovo; Rasim Beg bridge F.Y.R. Macedonia	2-1740
9	*Drossocarpium* Spach	*H. richeri* Vill. subsp. *grisebachii* (Boiss.) Nyman	Korab Mt., Kobilino pole, F.Y.R. Macedonia	21737
10		*H. barbatum* Jacq.	Bistra Mt., Medenica F.Y.R. Macedonia	1-1738

Mt.-mountin.

### 3.3. Extracts Preparation

The plant material was cut into small pieces, air-dried and extracted using a method of maceration with 70% of ethanol (EtOH) for 72 h at room temperature, according to the procedure given in EMA [3] and European Pharmacopoeia 6th Edition recommendations [42]. After the maceration, the extracts were collected, filtered and evaporated to dryness in a rotary evaporator. For evaluation of the total phenolic and flavonoid content, antioxidant activity and inhibition of acetylcholinesterase activity, the crude extracts were accurately weighed and dissolved in sufficient distilled water to produce10% (w/v) stock solutions. For chemical characterisation and quantification of the major pharmacologically active compounds (HPLC analysis), the dried extracts were dissolved in the mobile phase, to make 10% (w/v) stock solutions.

### 3.4. Determination of Total Phenolic Compounds and Flavonoid Content

The amount of total phenolic compounds in the extracts was determined colorimetrically with the Folin-Ciocalteu (FC) reagent using the method described before [33]. The concentration of total phenolic compounds was expressed as mg of gallic acid equivalents (GAE) per g of dried extract (de) (mg GAE/g de), using a standard curve of gallic acid. Measurement of the total flavonoid content in the investigated extracts was evaluated using the method based on the formation of complex flavonoid-aluminium with the absorptivity maximum at 430 nm [33]. The flavonoids content was expressed as mg of quercetin equivalents (QE) per g of dried extract (de) (mg QE/g de), using a standard curve of quercetin. All the measurements were carried out in five repetitions.

### 3.5. Chemical Characterisation by HPLC Analysis

Chemical characterisation of the examined extracts and quantification of the selected compounds was determined by high-performance liquid chromatography (HPLC). An Agilent HP 1100 HPLC-diode array detection (DAD) system equipped with an autosampler (Agilent, Waldbronn, Germany) was used. The components were separated using reversed-phase Zorbax CB-C18 column (4.6 × 150 mm, i.d., 5 μm particle size) held at 25 °C. Solvent A was 0.1% aqueous HCOOH with 10 mmol/L CH_3_COONH_4_, and solvent B was acetonitrile. The mobile phase used was delivered in the gradient mode (0 min 25% B, 6 min 100% B), with flow rate of 1 mL/min [35]. The HPLC mobile phase was prepared fresh daily and filtered through a 0.45 μm nylon filter. The injection volume was 10 µL. For the quantification of the selected compounds, standards (caffeic and chlorogenic acid, quercitrin, rutin, hyperforin and hypericin) were run under the same conditions, using solutions in methanol-water (50:50) (ranging 0.01–0.3 mg/mL) and detection was carried out at 270 and 590 nm.

### 3.6. Evaluation of Antioxidant Activity

Antioxidant properties of the examined extracts were evaluated, both for its free radical scavenging capacity (RSC) and for its protective effect on the process of lipid peroxidation (LP).

#### 3.6.1. Free Radical Scavenging Capacity (RSC)

*In vitro* RSC of the examined extracts was evaluated through a series of assays, in order to obtain relevant data concerning the antioxidant capacity of the ten different *Hypericum* extracts. One of the commonly used tests for screening RSC, the DPPH (2,2-diphenyl-1-pycrylhydrazyl) assay was applied as described before [33]. Tested concentrations of the investigated extracts ranged from 10.00 to 400.00 µg/mL. The disappearance of DPPH^·^ was measured spectrophotometrically at 515 nm.

The ability of the examined extracts (concentrations ranging from 10.00 to 400.00 µg of dry extract per mL) to neutralize the hydroxyl (OH) radical was determined by measuring the degradation of 2-deoxyribose with OH radicals, generated in a Fenton reaction. The degradation products were 2-thiobarbituric acid (TBA) reactive substances, which could be determined spectrophotometrically at 532 nm [32].

Selected concentrations (ranging from 10 to 500 µg/mL of de) of all the examined *Hypericum* species extracts were also tested for their ability to inhibit the nitric oxide (NO) release [35]. The reduction of NO^·^ in reaction with Griess reagent was measured spectrophotometrically at 546 nm.

For each sample in all the assays five replicates were recorded. The neutralization of the tested RSC, expressed in percentage, was calculated by the following Equation (1):

RSC (%) =100 × (*A_blank_* − *A_sample_*/*A_blank_*) (1)

#### 3.6.2. Inhibition of Lipid Peroxidation (LP)

The extent of LP was determined by measuring the colour of the adduct produced in the reaction between thiobarbituric acid (TBA) and malondialdehyde (MDA) in the TBA assay [33,43], performed with small modifications. Instead of liposomes “PRO-LIPO S”, as a model-system the commercial preparation of corn oil emulsified in phosphate buffer (0.035% v/v solution) with addition of 0.025% (v/v) Tween 80, was used. In the investigation, competition between the polyunsaturated fatty acids present in corn oil and neutralization of the OH radicals (induced in Fe^2+^/H_2_O_2_ system) by the extracts (concentrations ranging from 10.00 to 350.00 µg/mL) was measured. The content of MDA (thiobarbituric acid reactive species—TBARS) was determined by measuring the absorbance of the adduct at 532 nm. All the reactions were carried out in five repetitions.

The percentage of LP inhibition was calculated by the following Equation (2), where A_o_ was the absorbance of the control reaction (full reaction, without a test compound) and A_1_ was the absorbance of the examined samples:


I (%) = (A_o_ − A_1_)/A_o_*x* 100 (2)

### 3.7. Inhibition of Acetylcholinesterase Activity

The inhibition of acetylcholinesterase activity was determined spectrophotometrically by using acetylthiocholine iodide as substrate in modified Ellman method [44]. Briefly, sodium phosphate buffer (pH 7.2, 2.85 mL), colour indicator [0.15 mL, 5,5'-dithiobis-(2-nitrobenzoic acid)—DTNB containing NaHCO3], and the extracts sample (50 µL, concentrations ranging from 100.00 to 1,500.00 µg/mL) and acetylcholinesterase solution (10 µL, containing 2.37 U/L) were mixed in a tube and left at room temperature for 15 min. Subsequently, a solution of acetylthiocholine iodide (100 µL) was added. The hydrolysis of acetylthiocholine was determined by monitoring the formation of yellow 5-thio-2-nitrobenzoate anion as a result of the reaction of DTNB with thiocholine released by the enzymatic hydrolysis of acetylthiocholine at a wavelength of 405 nm. A control reaction was carried out using water instead of an extract and it was considered a 100% activity, while a blank with sodium phosphate buffer instead of enzyme solution was used. The tests were carried out in triplicate. The percentage of inhibition of acetylcholinesterase was calculated by the following Equation (3), where A_sample_ was the absorbance of the extract containing reaction mixture and A_control_ was the absorbance of the control reaction:


I (%) = 100 – (A_sample_/A_control_) × 100 (3)

### 3.8. Statistical Analyses

The data were reported as mean values ± standard deviation (SD). Values representing the concentrations of the investigated extracts that cause 50% of neutralization/inhibition (IC_50_) were determined by the regression analysis of the obtained RSC and the values of the inhibition of the LP or acetylcholinesterase activity inhibition (Microsoft Excel for Windows, v. 2007). The PCA analysis was done using Statistica, ver. 12.0 (Statosft Inc., Tulsa, OK, USA).

## 4. Conclusions

In general, the results of this study confirm that the geographical origin and the biological source can lead to notable changes in the chemical composition and the *in vitro* and consequently *in vivo* biological activity of the drug *Hyperici herba*, as well as in commercial preparations. Furthermore, some other *Hypericum* species, like *H. maculatum* subsp. *immaculatum*, which easily could be mixed up with *H. perforatum*, but also H*. olympicum*, *H. richeri* subsp. *grisebachii* and *H. barbatum,* which have exhibited strong antioxidant effects and inhibition of the acetylcholinesterase activity, should be further investigated for their *in vivo* pharmacological activities and as potential substances in preventing of Alzheimer’s disease. According to the high content of compounds used for the commercial preparations standardization (hypericin, hyperforin and total flavonoids), the most promising indigenous species is *H. barbatum*.

## Supplementary Materials

Supplementary File 1
