# Supplementary figures and images for: Impact of Origin and Biological Source on Chemical Composition, Anticholinesterase and Antioxidant Properties of Some St. John’s Wort Species (Hypericum spp., Hypericaceae) from the Central Balkans
Source: Molecules. 2013 Sep 25;18(10):11733–50. doi: 10.3390/molecules181011733 (PMC6270400; doi:10.3390/molecules181011733)

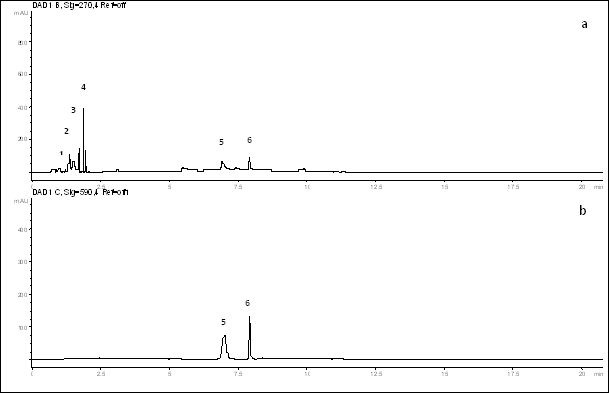


*Hypericum perforatum*


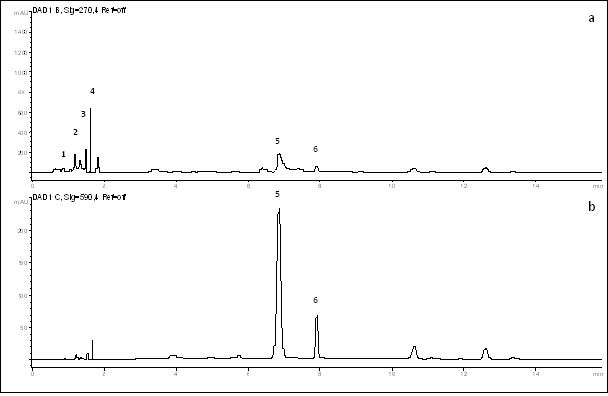


*Hypericum barbatum*

Supplement: Supplementary File 1 [file molecules-18-11733-s001.docx]
